# The effects of fibroblast growth factor-23 on diagnosis of cerebral infarction and vertebral basilar artery stenosis^☆^

**DOI:** 10.1016/j.clinsp.2024.100457

**Published:** 2024-08-02

**Authors:** Zhuoqun Wei, Changyang Zhong, Chunli Wu, Yuan Liu

**Affiliations:** a Zhejiang Chinese Medical University, Hangzhou, China; b Cerebrovascular Disease Department, Hangzhou Third People's Hospital, Hangzhou, China

**Keywords:** Fibroblast growth factor-23, Cerebral infarction, DSA, Stents, Vertebrobasilar artery stenosis

## Abstract

**Objectives:**

This study aimed to explore the correlation between Fibroblast Growth Factor-23 (FGF23) levels and Cerebral Infarction (CI), and to determine whether there is a significant relationship between FGF23 and the occurrence and severity of CI.

**Methods:**

The study categorized Cerebral Infarction (CI) patients into severe and mild stenosis groups based on vertebrobasilar artery stenosis, using Digital Subtraction Angiography (DSA) and Magnetic Resonance Imaging (MRI). The study compared the levels of Fibroblast Growth Factor-23 (FGF23) in the serum of CI patients and healthy controls using a *t*-test and evaluated the diagnostic effectiveness of serum FGF23 using a Receiver Operating Characteristic (ROC) curve. Additionally, the study analyzed the correlation between FGF23 levels and CI severity after treatment using the National Institute of Health Stroke Scale score.

**Results:**

The study found a significant increase in serum Fibroblast Growth Factor-23 (FGF23) levels in patients with Cerebral Infarction (CI) compared to healthy volunteers, (*p <* 0.001). A higher serum FGF23 level was observed in the severe stenosis group than in the mild stenosis group (*p <* 0.001). Furthermore, the study showed that a high FGF23 level at admission was significantly related to more severe symptoms of CI as indicated by the National Institute of Health Stroke Scale (NIHSS) score on the 7^th^ day after treatment (*p <* 0.001).

**Conclusions:**

This study discovered a correlation between Fibroblast Growth Factor-23 (FGF23) levels, vertebrobasilar artery stenosis, and short-term prognosis in patients who had recently experienced acute Cerebral Infarction (CI).

## Highlights

• A meta-analysis suggested that the risk for posterior-circulation stroke was roughly as high as the risk for anterior-circulation stroke. At the same time, the authors found that it was difficult to identify VBI upon routine physical examination with modalities such as carotid artery ultrasonography. The aim of this study was to investigate the possibility of FGF23 as a rapid biomarker for risk stratification of patients with acute CI and screen patients at high risk of CI.
• The study assessed the characteristics of patients with CI and found that the severity of VBI was a significant factor. Furthermore, serum FGF23 levels were significantly higher in patients with CI and significantly associated with NIHSS score on the 7th day after diagnosis. It is suggested that serum FGF23 level at admission may be a predictor of short-term outcomes in patients with CI.
• In the present study, FGF23 was a potential diagnostic indicator of CI with 79.8 % sensitivity and 80.3 % specificity. The ROC curve results indicated that serum FGF23 levels are a potential diagnostic biomarker for CI.
• This study provides important insights into the potential clinical utility of FGF23 as a diagnostic biomarker for CI and VBI.

Cerebral Infarction (CI) or ischemic stroke is a widespread cerebrovascular condition caused by the constriction of blood circulation in the brain due to thromboembolism and blockage of blood vessels.^1^ Various factors, such as hypertension, coronary heart disease, diabetes, smoking, and Vertebrobasilar Artery Stenosis (VBI), are recognized as risk factors for Cerebral Infarction (CI).^2^ In a previous study, the reported incidence of patients with VBI was as high as 10.9 %.^3^ The New England Medical Center Registry reported that 60 % of strokes in the posterior circulation were due to atherosclerosis.^4^ A meta-analysis suggested that the risk for posterior-circulation stroke was roughly as high as the risk for anterior-circulation stroke.^5^ At the same time, the authors found that it was difficult to identify VBI upon routine physical examination with modalities such as carotid artery ultrasonography. However, carotid stenosis itself is easily detected upon ultrasound examination. The clinical symptoms and sequelae of posterior circulation infarction are more serious,^6^ so it is necessary to further study the diagnosis and prevention of VBI and the CI caused by it.

In addition to the risk factors mentioned, a growing body of research has also indicated that high levels of Fibroblast Growth Factor-23 (FGF23), a hormone involved in regulating phosphate and vitamin D metabolism, may be associated with an increased risk of CI.^7^ One potential mechanism by which FGF23 may contribute to CI development is through its effects on vessel inflammation and endothelial dysfunction, which can ultimately lead to thromboembolism and embolic occlusion.^8^ Studies have found that endothelial dysfunction is mainly caused by reduced bioavailability of NO and increased generation of ROS. Endothelial dysfunction can lead to reduced vasodilation, enhanced vascular reactivity and atherosclerosis.^9^ Gross et al. established an *in vitro* model and found that moderate concentrations of FGF23 had the effect of stimulating aortic contraction and increasing ROS production by vascular smooth muscles, further leading to endothelial dysfunction.^10^ As early as 2009, Mirza et al. conducted a large-scale cohort study to explore the relationship between FGF23 and vascular endothelial function and atherosclerosis and found that elevated FGF23 was associated with impaired endothelium-dependent and endothelium-independent vasodilation in a natural population aged ≥ 70 years.^11^ Other studies have shown that FGF23 is an important mediator of vessel inflammation that can precede arteriolosclerosis or arterial stiffness, which are major causes of CI development.^12^ FGF23 is also involved in calcium-phosphate metabolism, and high levels of FGF23 may be associated with phosphate imbalance, which is a known risk factor for arterial stiffness and atherosclerosis.^13^

Vertebrobasilar artery stenosis is a major source of ischemic stroke. FGF23 may be a novel biochemical marker of inflammatory response in acute ischemia. However, to our knowledge, FGF23 has not yet been studied in the context of VBI. The aim of this study was to investigate the possibility of FGF23 as a rapid biomarker for risk stratification of patients with acute CI and screen patients at high risk of CI.

## Materials and methods

### Patient enrollment

The Ethics Committee of Hangzhou Third People's Hospital approved this study (IRB# 202011), and all participants provided informed consent. The authors confirm that informed consent was obtained from all participants and/or their legal guardians. These studies involving human research participants were all conducted in accordance with the guidelines of the Declaration of Helsinki. The study enrolled 80 patients (55 % male and 45 % female) with acute Cerebral Infarction (CI) from November 2020 to December 2022, with a mean age of 66.73 ± 7.12 years. The control group consisted of 80 healthy volunteers, with a mean age of 67.44 ± 7.75 years, including 52.5 % male and 47.5 % female.

All patients and healthy volunteers were diagnosed using physical examination, cranial Computed Tomography (CT), and Magnetic Resonance Imaging (MRI). Demographic and clinical data, such as age, sex, drinking and smoking history, presence of diabetes and/or hypertension, and Body Mass index (BMI), were collected from patients and healthy controls. Patients with a history of other cerebrovascular diseases such as hemorrhagic stroke or transient ischemic attack were excluded from the study. Patients who received treatment for CI, such as thrombolysis or endovascular intervention, were also excluded.

Inclusion criteria: 1) Confirmation of acute Cerebral Infarction (CI) at the posterior circulation based on MRI; 2) Adult patients (≥18-years); 3) Patients showing clinical signs of acute cerebrovascular disease, such as unilateral hemiplegia, aphasia, and unconsciousness, according to the National Institutes of Health Stroke Scale (NIHSS) criteria, within 6 hours of symptom onset and without a clear cause.

Exclusion criteria 1) Known history of hemorrhagic stroke or transient ischemic attack; 2) Recent treatment for CI, such as thrombolysis or endovascular intervention; 3) Severe liver or kidney disease; 4) Hypercalcemia or hyperparathyroidism; 5) Secondary hypertension due to renal artery stenosis or Cushing's Syndrome; 6) Pregnant or lactating status; and 7) Patients who had a contraindication to whole cerebral angiography were excluded from the study.

### Digital subtraction angiography and grouping

The severity of stenosis was determined by Digital Subtraction Angiography (DSA) according to standard guidelines. DSA is an invasive imaging technique that involves injecting a contrast medium into the bloodstream and using radiographic technology to create detailed images of the blood vessels in the brain. DSA is considered the gold standard for diagnosing cerebral arterial stenosis, and it is commonly used in patients with CI to identify the location and severity of arterial stenosis. Patients with acute CI were divided into two groups based on the severity of stenosis in the vertebrobasilar artery. The severity of stenosis was determined using DSA according to standard guidelines. Patients with > 50 % stenosis of the vertebrobasilar artery were defined as having severe stenosis, while those with < 50 % stenosis of the vertebrobasilar artery were defined as having mild stenosis.

### Data collection

Blood samples were collected from each patient within 24h of admission and were used to measure the FGF23 levels using Enzyme-Linked Immunosorbent Assay (ELISA) kits. The conditions of patients after treatment were assessed based on the NIHSS criteria. Furthermore, the study investigated the association between serum FGF23 levels at admission and the post-treatment National Institutes of Health Stroke Scale (NIHSS) score.

### Detection of serum FGF23 levels

Peripheral venous blood samples were obtained from both patients and healthy controls and analyzed using a double-antibody, two-step, sandwich enzyme-linked immunosorbent assay (ELISA). FGF23 kits were purchased from R&D systems, USA.

The steps are as follows: Leave all items at room temperature for 15 minutes, mix gently, and the working diluents should be mixed on the spot.

1. Embedding antibodies:
1.1) The Capture antibody was diluted to a certain working concentration in dilution buffer (PBS) without carrier protein;

Degree (Capture + 0.5 mL PBS), and immediately diluted Capture antibodies were uniformly plated on each 96-well plate;

On the top, 100 μL could be inhaled into each well, the well plate was sealed and incubated at room temperature overnight.

1.2) The Capture antibody was aspirated out and washed with Wash Buffer at 400 μL/well for a total of 3. After each cleaning, the residual liquid was removed, and after the last cleaning, the residual liquid in the hole was sucked with a clean paper towel Wash Buffer.
1.3) Add 300 μL Reagent Diluent (diluted to 1 × with PBS) to each well in turn, The plates were blocked and incubated for 1 hour at room temperature.
1.4) Repeat step 2; The antibody was successfully embedded.
2. Prepare for testing:
2.1) Add 100 μL of diluted sample (diluted using 1 × RD) or diluted standard, cover with tape, in the cells were incubated for 2 hours at room temperature.
2.2) Repeat step 2 of the antibody embedding procedure.
2.3) Add 100 µL of Detection Antibody (DA+1 ml RD), cover the tape, and keep at room temperature the cells were incubated for 2 hours.
2.4) Repeat step 2 of the antibody embedding procedure.
2.5) 100 μL Streptavidin-HRP working dilution was added to each well and placed at room temperature to incubate for 20 minutes;
2.6) Repeat step 2 of the antibody embedding procedure.
2.7) Add 100 μL Substrate Solution (Color Reagent A + Color Reagent B = 1:1) to each well, the cells were incubated at room temperature for 20 minutes, avoiding direct exposure to light.
2.8) Evenly add 50 μL Stop Solution to each well and shake the plate gently to make it fully mixed Uniform.
2.9) The absorbance in each well was measured by a microplate reader with a wavelength of 450 nm to obtain the OD value.
2.10) Using the OD value of the sample as the abscissa and the standard concentration as the ordinate, through the OD value and the standard concentration the curve regression equation can be obtained by the degree, and the OD value of the sample can be substituted into the regression equation to finally obtain the serum Corresponding concentrations of FGF23.

### Detection of biochemical indicators

The study's clinical laboratory used an automated biochemical analyzer (Architect Plus C8000, Abbott, USA) to detect biochemical indicators such as Triglycerides (TG), Total Cholesterol (TC), High-Density Lipoprotein Cholesterol (HDL-C), and Low-Density Lipoprotein Cholesterol (LDL-C).

### Statistical analysis

Data were analyzed using SPSS software (version 22.0; IBM Corporation, Armonk, NY, USA). Continuous variables were expressed as means ± Standard Deviation (SD), and categorical variables were expressed as frequencies and percentages. The distribution of variables was checked for normality using the Kolmogorov-Smirnov test. Comparisons between groups were performed using *t*-tests or Mann-Whitney *U* tests for continuous variables and Chi-Squared tests or Fisher's exact tests for categorical variables. Multiple linear regression analysis was used to assess the association between FGF23 levels and NIHSS scores after adjusting for potentially confounding variables. A p-value of < 0.05 was considered to indicate statistically significant differences.

## Results

### Characteristics of patients with CI

Table 1 provides an overview of the baseline and clinical characteristics of 80 patients with Cerebral Infarction (CI) and 80 healthy volunteers. Out of the 80 patients, 46 had severe vertebral-basilar insufficiency (> 50 %) and 34 had mild VBI (< 50 %). No significant differences were observed in age, sex, BMI, TC, TG, HDL-C, and LDL-C between patients with CI and healthy volunteers (all p > 0.05).

**Table 1:** The detailed clinical information of healthy volunteers and cerebral infarction patients (Mean ± SD).

Characters	Mild stenosis (*n* = 34)	Severe stenosis (*n* = 46)	Healthy volunteers
Gender			
Male	24	20	42
Female	10	26	38
Age	65.84 ± 7.93	68.14 ± 7.95	67.44 ± 7.75
Hypertension	9	29	/
Smoking	10	21	33
Diabetes	12	30	/
BMI (Kg/m^2^)	23.12 ± 2.56	24.68 ± 2.11	23.63 ± 2.08
HDL	1.54 ± 0.41	1.76 ± 0.22	1.46 ± 0.72
TC (mmoL/L)	4.50 ± 1.31	4.89 ± 0.79	4.69 ± 0.62
IL6 (pg/mL)	4.41 ± 1.19	4.73 ± 1.09	4.53 ± 1.06
TG (mmoL/L)	5.82 ± 0.69	6.18 ± 0.77	6.04 ± 0.57
Glycosylated hemoglobin	6.43 ± 2.13	7.03 ± 2.06	6.81 ± 2.04
LDL (mmoL/L)	2.13 ± 0.59	4.29 ± 0.66	3.17 ± 0.67

There were no significant differences in age, gender, hypertension and other indicators between the two groups (p > 0.05).

### Serum FGF23 levels

**Fig. 1:**
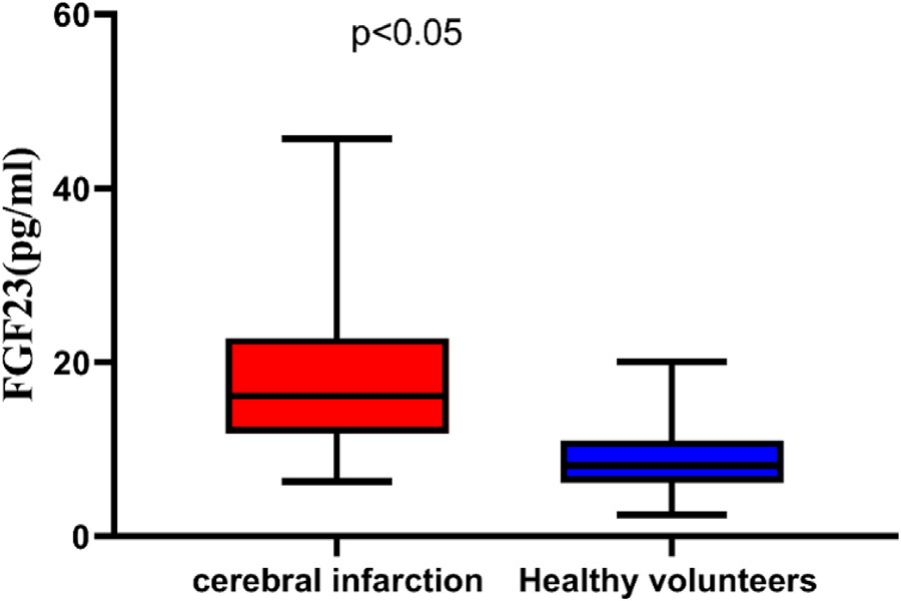
Patients with Cerebral Infarction (CI) exhibited significantly higher serum FGF23 levels than the healthy volunteers.

**Fig. 2:**
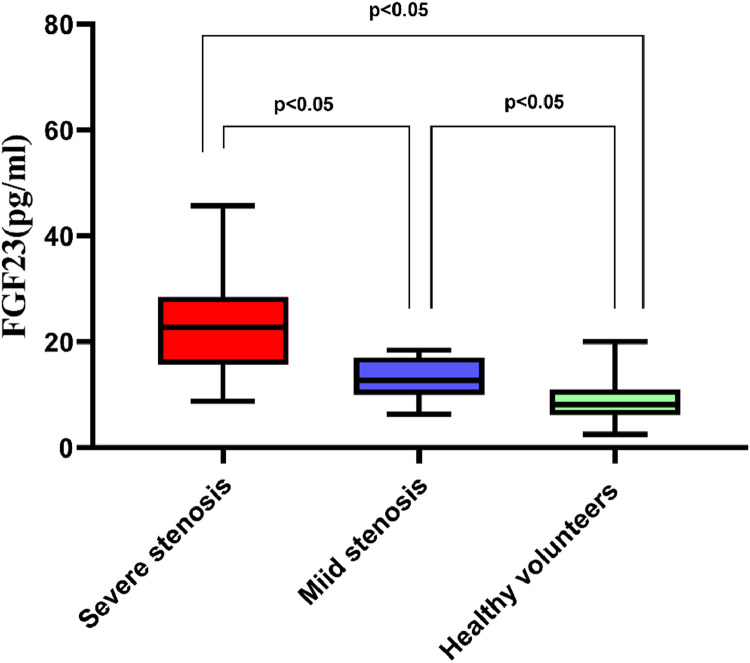
Serum FGF23 levels in patients with severe or mild stenosis was higher than those in healthy volunteers, serum FGF23 levels in severe stenosis group was increased compared to mild stenosis group.

Fig. 1 demonstrates that patients with Cerebral Infarction (CI) had significantly higher serum FGF23 levels compared to healthy volunteers (18.53 ± 8.19 pg/mL vs. 8.57 ± 3.45 pg/mL, respectively; *p <* 0.05, 95 % CI 7.247–11.23). Moreover, Fig. 2 indicates that serum FGF23 levels were significantly higher in patients with severe stenosis than in those with mild stenosis (22.66 ± 9.24 pg/mL vs. 13.47 ± 3.83 pg/mL, respectively; *p <* 0.05, 95 % CI 5.253–13.13).

### ROC curve results

**Fig. 3:**
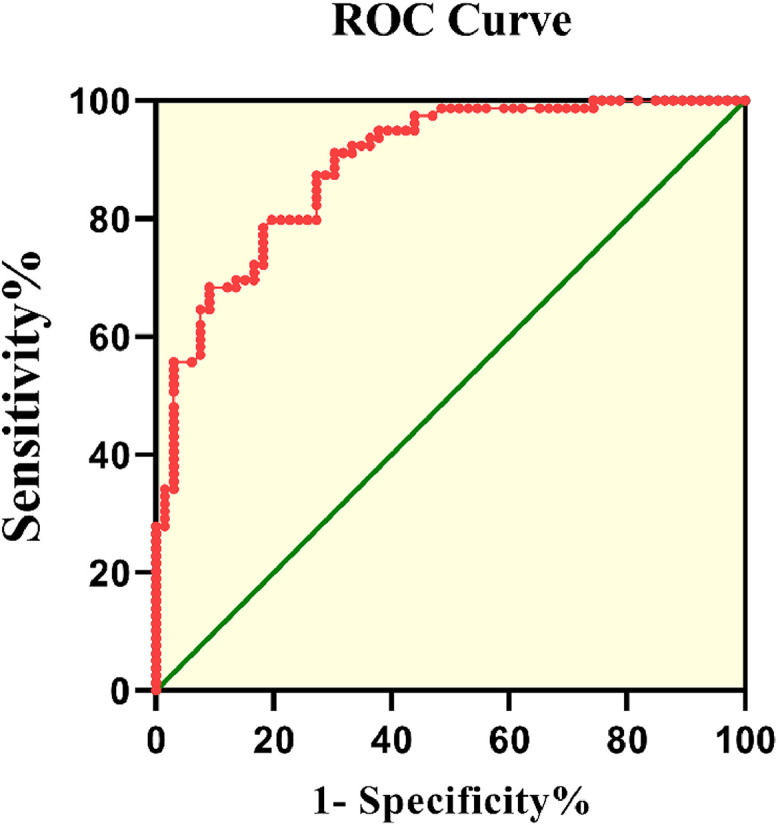
ROC curve of FGF23 level for CI. The Area Under the Curve (AUC) value was 0.89 (95 % CI 0.839–0.941, *p <* 0.001).

Fig. 3 displays the ROC curve of FGF23 levels for patients with Cerebral Infarction (CI). The Area Under the Curve (AUC) value was 0.89 (95 % CI 0.839–0.941, *p <* 0.001). According to the ROC curve, the cut-off value of serum FGF23 levels for patients with CI was 11.2 pg/mL, with 79.8 % sensitivity and 80.3 % specificity.

### Correlation between NIHSS score and FGF23 level

On the 7^th^ day after treatment, all patients with acute CI were assessed based on the NIHSS criteria. The results of the NIHSS score and serum FGF23 levels are shown in Table 2. Serum FGF23 levels at admission were associated with the NIHSS score on the 7^th^ day after diagnosis (*p <* 0.05).

**Table 2:** Before and after 14 days of treatment, serum FGF23 level in each group were correlated with NIHSS.

NIHSS score (point)	1‒4	5‒15	≥16	p
**On admission**				
Patient (case)	26	30	24	
Serum FGF23 (pg/mL)	14.72 ± 2.62	18.66 ± 6.41	25.58 ± 7.01	<0.05
**14 days after treatment**				
Patient (case)	38	27	15	
Serum FGF23 (pg/mL)	13.18±2.13	17.32±6.72	24.01±7.02	<0.05

## Discussion

The prognosis of ischemic stroke is usually debilitating. Early diagnosis and treatment of ischemic stroke are vital for good prognosis. Many studies have been carried out, but the results are all unsatisfactory and seem hopeless. In this context, this study investigated the effects of FGF23 on the diagnosis of CI and VBI. The early identification of biomarkers for ischemic stroke diagnosis is crucial.

Fibroblast growth factor-23 is a biomarker involved in the regulation of phosphate and vitamin D metabolism. Fibroblast growth factor is a signaling protein with a variety of biological activities that are activated by binding to its receptor (FGFR). It mediates several biological effects such as mitosis, cell differentiation and migration, angiogenesis, and tissue injury repair.^14^ At present, there are four known FGFR (FGFR 1–4), which mainly exert biological effects by activating the RAS-MAPK and PI3K-AKT pathways. The FGF family consists of 22 structurally similar proteins, FGF1–FGF23, which are absent in humans and mice, respectively, because FGF15 and FGF19 are homologous proteins.^15^ The FGF23 gene is located on human chromosome 12 and mouse chromosome 6 and comprises three exons and two introns. It is a 32 KDa glycoprotein. The main functions of FGF23 are to reduce renal phosphate reabsorption and suppress circulating levels of 1.25-(OH)2D3. FGF23 is a pathogenic gene of autosomal dominant hypophosphatemic rickets,^16^ and it is also a circulating factor in tumor-induced osteomalacia. At the same time, some studies have found that Klotho, FGFR1, and FGFR3 are expressed in the vascular wall, which suggests that the vascular wall is the target organ of FGF23, and also that FGF23 is involved in the occurrence and development of cardiovascular diseases.^17^

Based on the study findings, serum FGF23 levels were elevated in patients with Cerebral Infarction (CI) and Vertebral-Basilar Insufficiency (VBI), suggesting the potential use of serum FGF23 levels for diagnosing CI. Previous studies have shown that elevated serum levels of FGF23 are associated with an increased risk of cardiovascular disease, including CI.^18^ The study by Scialla et al. has similarly shown that elevated FGF23 levels may be associated with an increased risk of cardiovascular disease and stroke.^19^ However, the present study specifically investigated posterior circulation infarction and showed, for the first time, that FGF23 levels were significantly higher in patients with CI caused by VBI than in healthy controls. To the best of our acknowledge, this is the first study to report a positive correlation between serum FGF23 levels and VBI. On this basis, the present study further revealed that serum FGF23 levels were significantly higher in patients with CI and VBI than healthy controls. Moreover, the prevalence of VBI was significantly higher in patients with high FGF23 levels than in those with low FGF23 levels. These findings suggest that serum FGF23 may be useful as a diagnostic biomarker for CI and VBI. This finding provides a basis for understanding the disease evolution of patients with CI and the early diagnosis of severe VBI, and provides a reference for adjusting the early treatment plan in VBI. Early intervention and regular monitoring of patients with elevated FGF23 levels can help alleviate the progression of the disease and improve their quality of life. The present findings expand upon this evidence by demonstrating the potential usefulness of FGF23 as a diagnostic biomarker for CI.

The study assessed the characteristics of patients with CI and found that the severity of VBI was a significant factor. Furthermore, serum FGF23 levels were significantly higher in patients with CI and significantly associated with NIHSS score on the 7^th^ day after diagnosis. It is suggested that serum FGF23 level at admission may be a predictor of short-term outcomes in patients with CI.

In the present study, FGF23 was a potential diagnostic indicator of CI with 79.8 % sensitivity and 80.3 % specificity. The ROC curve results indicated that serum FGF23 levels are a potential diagnostic biomarker for CI.

This study provides important insights into the potential clinical utility of FGF23 as a diagnostic biomarker for CI and VBI. Nevertheless, this study has some limitations. First, it was a single-center observational study, and the sample size of this study was small. Additionally, the study did not evaluate confounding factors such as hypertension or diabetes, which may influence serum FGF23 levels and its association with CI and VBI. Therefore, further research is needed to address these limitations in the investigation of the potential role of FGF23 in the diagnosis and treatment of cerebrovascular disease.

In summary, the findings from this study provide important insights into the potential clinical utility of FGF23 as a biomarker for CI and its association with VBI and stroke severity. Further research is needed to confirm the findings and investigate the potential therapeutic implications of targeting FGF23 in the prevention and treatment of cerebrovascular disease.

## Conclusions

The study indicates that serum FGF23 level is correlated with vertebrobasilar artery stenosis and may serve as a potential biomarker for diagnosing Cerebral Infarction (CI).

## Compliance with ethical standards

The Hangzhou Third People's Hospital approved the study, and all participants provided informed consent.

## Patient permission/consent declarations

All patients signed an informed consent approved by the Institutional Review Board.

## Declaration of competing interest

The authors declare that the research was conducted in the absence of any commercial or financial relationships that could be construed as a potential conflict of interest.
